# The Effects of Long-Term Graft Preservation on Intraoperative Hemostatic Changes in Liver Transplantation

**DOI:** 10.1155/1994/27915

**Published:** 1994

**Authors:** C. Minke Bakker, Jan D. Blankensteijn, Peter Schlejen, Robert J. Porte, Maria J. Gomes, Harald I. H. Lampe, Jeanne Stibbe, Onno T. Terpstra

**Affiliations:** 1 Department of Internal Medicine University Hospital Dijkzigt Erasmus University Rotterdam The Netherlands; 2 Department of Surgery University Hospital Dijkzigt Erasmus University Rotterdam The Netherlands; 3 Department of Hematology University Hospital Dijkzigt Erasmus University Rotterdam The Netherlands

## Abstract

We compared hemostatic changes during OLT and HLT after various periods of graft storage, to investigate whether the host liver in HLT protects the recipient from hemostatic deterioration induced by severe graft storage damage. In particular, the mechanism of fibrinolytic deterioration was investigated. The effect of prostaglandin E_1_ (PGE_1_) on these parameters was also studied.

*Material and Methods*: 69 pigs underwent either OLT (N = 32) or HLT (N = 37) with a graft stored for 2 hr (N = 31), 24 hr (N = 16), 48 hr (N = 7), or 72 hr (N = 15). PGE_1_ was given intravenously to both donor and recipient animals and was added to the preservation and flushing solutions. Fibrinolysis (euglobulin clot lysis time, t-PA activity and *α*_2_-antiplasmin) and coagulation parameters (activated partial thromboplasmin time, prothrombin time, fibrinogen and platelet count) were measured at several intervals during transplantation.

*Statistics*: Univariate non-parametric tests were used for analysis of coagulation and fibrinolysis parameters. For the three main variables- i.e., the type of transplantation, the use of PGE_1_, and the preservation time, multiple regression analysis was performed.

*Results*: Fibrinolytic activity increased during the anhepatic period of OLT. Graft reperfusion was followed by a rise in t-PA in both OLT and HLT. In HLT, t-PA quickly returned to normal, whereas a continuous increase was found in OLT. The coagulation parameters, in turn, remained unchanged during the anhepatic period and deteriorated in OLT compared to HLT. The duration of graft storage was directly related to the severity of the hemostatic changes, although this effect was more apparent in OLT than in HLT.

*Conclusions*: Changes in hemostasis are more pronounced in OLT than in HLT. This suggests that the host liver protects the recipient from the effects of graft storage damage, even after long preservation times. Early postreperfusion fibrinolytic activity was presumably due to t-PA release from the graft both in OLT and HLT. The further rise t-PA in OLT might be caused by the release of cytokines from the graft, that subsequently evoke endothelial t-PA release. In HLT, t-PA and cytokines may be cleared by the native liver. No positive or negative effect of PGE_1_ on coagulation or fibrinolysis parameters was noticed.


*HPB Surgery*, 1994, Vol. 7, pp. 265–280
Reprints available directly from the publisher
Photocopying permitted by license only

© 1994 Harwood Academic Publishers GmbH
Printed in the United States of America

### THE EFFECTS OF LONG-TERM GRAFT PRESERVATION ON INTRAOPERATIVE HEMOSTATIC CHANGES IN LIVER TRANSPLANTATION

### A Comparison Between Orthotopic and Heterotopic Transplantation in the Pig

C. MINKE BAKKER[1], JAN D. BLANKENSTEIJN[2], PETER SCHLEJEN[2], ROBERT J. PORTE[1], MARIA J. GOMES[3], HARALD I.H. LAMPE[2], JEANNE STIBBE[3] and ONNO T. TERPSTRA[2]

*Departments of Internal Medicine[1], Surgery[2], and Hematology[3] University Hospital Dijkzigt, Erasmus University, Rotterdam, The Netherlands*

We compared hemostatic changes during OLT and HLT after various periods of graft storage, to investigate whether the host liver in HLT protects the recipient from hemostatic deterioration induced by severe graft storage damage. In particular, the mechanism of fibrinolytic deterioration was investigated. The effect of prostaglandin $E_1$ ($PGE_1$) on these parameters was also studied.

*Material and Methods*: 69 pigs underwent either OLT (N = 32) or HLT (N = 37) with a graft stored for 2 hr (N = 31), 24 hr (N = 16), 48 hr (N = 7), or 72 hr (N = 15). $PGE_1$ was given intravenously to both donor and recipient animals and was added to the preservation and flushing solutions. Fibrinolysis (euglobulin clot lysis time, t-PA activity and $\alpha_2$-antiplasmin) and coagulation parameters (activated partial thromboplasmin time, prothrombin time, fibrinogen and platelet count) were measured at several intervals during transplantation.

*Statistics*: Univariate non-parametric tests were used for analysis of coagulation and fibrinolysis parameters. For the three main variables- i.e., the type of transplantation, the use of $PGE_1$, and the preservation time, multiple regression analysis was performed.

*Results*: Fibrinolytic activity increased during the anhepatic period of OLT. Graft reperfusion was followed by a rise in t-PA in both OLT and HLT. In HLT, t-PA quickly returned to normal, whereas a continuous increase was found in OLT. The coagulation parameters, in turn, remained unchanged during the anhepatic period and deteriorated in OLT compared to HLT. The duration of graft storage was directly related to the severity of the hemostatic changes, although this effect was more apparent in OLT than in HLT.

*Conclusions*: Changes in hemostasis are more pronounced in OLT than in HLT. This suggests that the host liver protects the recipient from the effects of graft storage damage, even after long preservation times. Early postreperfusion fibrinolytic activity was presumably due to t-PA release from the graft both in OLT and HLT. The further rise t-PA in OLT might be caused by the release of cytokines from the graft, that subsequently evoke endothelial t-PA release. In HLT, t-PA and cytokines may be cleared by the native liver. No positive or negative effect of $PGE_1$ on coagulation or fibrinolysis parameters was noticed.

KEY WORDS: Liver transplantation, fibrinolysis, DIC, PGE

### INTRODUCTION

Orthotopic liver transplantation (OLT) has become an accepted method to treat patients with end-stage chronic liver disease. However, the procedure is frequently complicated by severe changes in hemostasis. Both increased fibrinolysis[1,2] and disseminated intravascular coagulation[3,4,] have been implicated in playing a role. The most striking abnormalities occur late in the anhepatic period and become more marked after reperfusion of the graft[5,6]. Earlier studies have demonstrated that the severity and duration of hemostasis abnormalities were mainly related to the quality of the donor liver[7,8].Release of activated hemostasis factors and/or humoral substances from the graft, that may interfere with hemostasis have been suggested to play a role.

Auxiliary heterotopic liver transplantation (HLT) has been proposed as an alternative to hepatic replacement. In HLT, the host liver is left *in situ* and the graft is transplanted in a heterotopic position. The anhepatic period is avoided and the function of the host liver retained. Hence, substances released from the allograft at reperfusion might be cleared and the deterioration of hemostasis less severe. Recently, we demonstrated that changes in fibrinolysis after reperfusion and more severe and substained in OLT than in HLT after 2 hours of simple cold graft storage[9]. This suggested that the native liver in HLT protects the recipient from the changes induced by preservation damage. We were now interested in whether the host liver in HLT also protects the recipient from the effects of long-term preserved grafts as well as the mechanism of hemostatic deterioration. We have thereby concentrated on fibrinolysis and included, additionally to previous studies, t-PA activity measurement.

Graft protection by addition of prostaglandin $E_1$ ($PGE_1$) to the flush solution has been demonstrated in human liver transplantation[10]. Furthermore, successful liver transplantations have been reported after long-term simple cold storage with University of Wisconsin (UW) solution without addition of prostaglandins: for 24 hours and more in man[11] and for 48 hours in dogs[12].It is not known if cytoprotective agents give additional protection. Therefore we also studied the effects on hemostasis of adding of $PGE_1$ to the preservation fluid.

### METHODS

Sixty-nine female Yorkshire pigs were randomly allocated to OLT (N = 32) or HLT (N = 37). Thirty-one grafts were transplanted after 2 hr of cold storage, 16 after 24 hr, 7 after 48 hr and 15 after 72 hr. In the 2-hr experiments, Eurocollins' solution was used for graft storage because UW solution was not yet available. In the long-term preservation experiments (24-hr, 48-hr, and 72-hr), UW solution was used. Sixteen transplantations in the different preservation groups were performed using $PGE_1$ (Table 1). Body weights of the donor and recipient animals were similar, about 25 Kg. Histocompatibility was matched by the mixed lymphocyte culture-test[13].

### *Surgical Technique*

Donor and recipient operations were performed as described earlier[14]. In OLT, a venovenous heparin-coated iliacoportajugular bypass was introduced[15].

**Table 1** Number of animals in the various preservation groups

| | *Experimental Group* | | | | | | | | *Total* |
|---|---|---|---|---|---|---|---|---|---|
| | 2 hr[a] | | 24 hr | | 48 hr | | 72 hr | | |
| | OLT | HLT | OLT | HLT | OLT | HLT | OLT | HLT | |
| PGE, + | 0 | 0 | 4 | 4 | 1 | 2 | 1 | 4 | 16 |
| PGE, − | 16 | 15 | 4 | 4 | 1 | 3 | 5 | 5 | 53 |
| Total | 16 | 15 | 8 | 8 | 2 | 5 | 6 | 9 | 69 |

[a]Duration of liver preservation.

Blood loss was estimated from the amount of liquid sucked away from the surgical field and collected in Buleaux bottles during the transplantation. Depending on the amount of blood loss, 500–1000 ml donor blood was given to the recipient. Usually, this was necessary around 30 minutes after graft reperfusion. Anesthetic techniques were the same for all animals.

### *Prostaglandin*

When $PGE_1$ was given, this was started 60 min before donor hepatectomy by an intravenous infusion rate of 100 ng/kg/min[16]. In addition, 1 mg/L $PGE_1$ was added to the UW solution[10]. The recipient animal was treated with a similar infusion as the donor, starting about 60 min before the recirculation of the graft. Before implantation, the graft was rinsed via the portal vein with two liters of cold (4° C) 0.9% saline to which 1 mg/L $PGE_1$ was added. $PGE_1$ was supplied by The Upjohn Company, The Netherlands.

### *Blood Samples*

For comparison, the moments of measurement were synchronized, relative to the moment of recirculation. Blood samples were drawn around every important operative step (Table 2) to assess hemoglobin levels, platelet counts, and various hemostasis parameters. Additionally, on reperfusion, the first 30 ml of perfusate were drawn from the infrahepatic vena cava of the graft. These first hepatic outflow samples (FR) after reperfusion were also subjected to hemostasis studies.

In OLT, R-5 is a measurement in the anhepatic phase. In HLT, there is no real anhepatic phase, but during the portal vein anastomosis there is a period of partial clamping of the portal vein. In the pig this often implies (almost) complete clamping.

**Table 2** Intraoperative moments of hemostatic measurements

| | |
|---|---|
| R-60 | Starting value: ± 1 hr before recirculation. |
| R-5 | Anhepatic phase: ± 5 min before recirculation. |
| FR | First hepatic outflow after reperfusion. |
| R+5 | ± 5 min after recirculation. |
| R+30 | ± 30 min after recirculation (during the construction of the arterial anastomosis). |
| R+60 | ± 60 min after recirculation (5 min after the aorta anastomosis). |
| R+90 | ± 90 min after recirculation (closure). |

Blood samples (20 ml) were taken from an arterial line. Eighteen ml of blood was divided equally into two polystyrene test tubes, containing 1 ml ice-cold trisodium citrate 0.11 mol/l and immediately placed on melting ice. Plasma was collected after centrifugation (2800 g, 4° C, 20 min), snap-frozen and stored in small aliquots at -70° C until used. Two ml blood was also collected into 0.045 ml 15% sol 6.75 mg EDTA.

A normal plasma pool was prepared from equal volume samples obtained from 10 animals immediately after induction of anaesthesia. This pool was defined as having 100% of normal coagulation and fibrinolysis proteins.

### *Hemostasis Studies*

Tissue-type plasminogen activator (t-PA) was assayed according to Verheijen *et al.*[17] using plasminogen, t-PA stimulator and S-2251 (from Kabi Diagnostica, Woerden, The Netherlands) and anti (porcine-heart) t-PA immunoglobulin (from Biopool AB, Umea, Sweden). To determine the euglobulin clot lysis time (ECLT), standard euglobulin fractions of plasma were prepared at pH 5.9 with a plasma dilution of 1:10[18]. Precipitates were redissolved in Tris/Tween buffer (0.1 M Tris/HCl, containing 0.1% Tween 80 [v/v] pH 7.5) and to 0.2 ml aliquots of the dissolved euglobulin fractions 0.1 ml portions of calcium-thrombin solution ($CaCl_2$ 25 mmol/l and thrombin 10 NIH U/l) were added to induce clot formation. The lysis time of the clot was recorded. The disappearance of air bubbles was regarded as the endpoint of lysis. $\alpha_2$-Antiplasmin ($\alpha_2$-AP) activity was measured according to Friberger *et al.*[19] using Coatest* antiplasmin (Kabi Diagnostica, Woerden, The Netherlands). Fibrinogen was measured according to Clauss[20] using thrombin from DiaLab, Leusden, The Netherlands.

Routine clotting times, including the activated partial thromboplastin time (aPTT), reflecting the intrinsic coagulation pathway and the prothrombin time (PT), reflecting the extrinsic pathway, performed according to standard procedures. Reagents used were Actin* (Baxter Dade AG, Düdingen, Switzerland) for the aPTT and Thromborel-S* (Behring Diagnostica, Amsterdam, The Netherlands) for the PT.

### *Statistics*

Data were subjected to computerized statistical analysis (PATFILE statistical package). Continuous variables are given as median (mean, ± standard error of the mean) and analyzed by nonparametric tests: in case of independent samples the Mann-Whitney test was used, and in case of paired samples the Wilcoxon rank-sum test. The coagulation changes were also analyzed in a multiple regression model (SPSS/PC+) using the three main variables in this study: the type of transplantation, the use of $PGE_1$ and the preservation time. The relative influence of these variables on a coagulation parameter is represented by the T-value (ratio between B and Standard Error of B) together with the significance of T.

### RESULTS

Meaningful changes in hemostasis parameters during the operation are elaborated on in the following paragraph. Data are presented separately for each of the three main variables in this study: the type of transplantation, the effect of graft preservation time, and the effect of $PGE_1$.

### *Type of Transplantation*

*Fibrinolysis parameters:* The changes in t-PA activity were reciprocal to those in ECLT. During OLT, t-PA activity levels rose significantly during the anhepatic period ($p < 0.02$), and increased sharply after reperfusion ($p < 0.05$) till the end of the operation. In contrast, during HLT, t-PA activity levels remained low before reperfusion, increased temporarily after reperfusion ($p < 0.01$) and normalized again thereafter. The proportional increase in t-PA activity was similar in HLT and OLT immediately after reperfusion. At all sampling points, except R-60 and R + 5, the t-PA activity in OLT was significantly higher than in HLT (at R-5: T = -2.20, $p = 0.03$ and at R + 30 through R + 90: T between -2.79 and -3.12, $p < 0.01$).

The t-PA activity levels were higher in the first venous outflow of the graft (FR) in HLT (1.22 IU/ml, median value) compared to OLT (0.36 IU/ml, $p < 0.02$), while this difference was not yet present in the portal vein blood entering the graft (T = 1.06, $p = 0.32$).

$\alpha_2$-AP levels declined significantly from R-60 through R + 5 ($p < 0.001$), and remained almost unchanged thereafter. $\alpha_2$-AP levels were lower in OLT than in HLT in the postreperfusion period from R + 30 through R + 90 (T between 2.08 and 3.37, $p < 0.05$).

*Coagulation parameters:* The PT and aPTT increased slightly in the period before reperfusion and increased significantly thereafter ($p < 0.001$). Before reperfusion and immediately thereafter, there was no significant difference between OLT and HLT for these tests, but in the postreperfusion period, at R + 30 and R + 60, the PT (T = -3.48 and T = -3.55, $p = 0.001$) and aPTT (T = -3.83 and T = -4.11, $p < 0.001$) were prolonged in OLT compared to HLT.

Fibrinogen levels practically halved before reperfusion ($p < 0.001$) without reaching a significant difference between both types of transplantation ($p = 0.98$). After reperfusion, fibrinogen levels remained stable and at R + 5 and R + 30 the fibrinogen level in OLT was significantly lower compared to HLT (T = 2.05 and T = 2.09, $p < 0.05$).

### *Graft Storage Time*

*Fibrinolysis parameters:* The preservation time had an evident effect on the fibrinolysis parameters, although not yet apparent at R + 5 (for t-PA: T = 0.45, $p = 0.66$). Especially at R + 60 and R + 90, the t-PA activity (Figure 1), the ECLT and $\alpha_2$-AP deteriorated with increasing preservation times. The cold storage time had some effect on t-PA activity and ECLT before reperfusion, although these did not reach statistical significance in the multiple regression analysis (T = 0.88, P = 0.39 and T = -0.86, $p = 0.39$). The first venous outflow (FR) from the 2-hr and 24-hr preserved grafts contained high levels of t-PA activity. No increased t-PA levels could be demonstrated in the first venous outflow from the 48-hr and 72-hr preserved grafts.

*Coagulation parameters:* The multiple regression analysis for the effect of the graft storage time revealed a clear influence on the PT and aPTT levels (Figures 2 and 3, respectively). This was evident in the period after reperfusion, starting at R

+ 5 (T = 2.38, P = 0.02 and T = 3.88, $p < 0.001$) and continuing till R + 30 for PT (T = 3.17, $p < 0.01$) and R + 60 for aPTT (T = 3.93, $p < 0.001$).

In contrast, fibrinogen levels were not significantly influenced by graft storage time. At R + 5 and R + 30 a longer ischemic period correlated with a decreased fibrinogen level, but the difference was not significant.

The preservation time had an evident effect on the platelet count (all T-values below -3.0 and $p < 0.005$).

<B>Use of PGE

Neither coagulation nor fibrinolysis tests were affected by $PGE_1$ administration at any of the measured points (data not shown).

### *Blood Loss and Survival*

The amount of blood loss and blood transfusion in the different groups are given in Table 3. In the multiple regression analysis blood loss was significantly controlled by the type of transplantation (T = -2.33, $p = 0.02$) and the preservation time (T = 2.88, $p < 0.01$). Because none of the animals survived OLT after 72 hr graft storage, many never received a second unit of blood. This explains why it appeared that HLT required more blood transfusion in the 72 hr experiment despite the higher blood loss in OLT. In the multiple regression the duration of preservation was a significant determinant of survival (T = -3.59, $p < 0.001$). Survival was not significantly different in the 2 hr preservation experiments. However, in the 24 hr and 48 hr experiments, survival was significantly longer after HLT compared to OLT ($p < 0.05$). Survival in the extended preservation experiments was dismal: In OLT all animals died the day of the operation after 48 hr and 72 hr graft preservation (three of which died within 30 minutes after reperfusion) and in HLT all animals after 72 hr graft preservation. Three animals survived more than 3 weeks after HLT of a 24 hr preserved graft. Two more animals survived after HLT for two days, one in the 24 hr group and one in the 48 hr group. In Table 4 the causes of mortality are categorized.

**Table 3** Median blood loss and blood transfusion during the transplantation

| | *2 hr* | | *24 hr* | | *48[c] hr* | | *72 hr* | |
|---|---|---|---|---|---|---|---|---|
| | *OLT* | *HLT* | *OLT* | *HLT* | *OLT* | *HLT* | *OLT* | *HLT* |
| **Blood loss** ml | | | | | | | | |
| —median | 400 | 500[a] | 850 | 425[b] | 160 | 1125 | 1100 | 925[b] |
| —lowest | 250 | 250 | 400 | 100 | | 700 | 600 | 200 |
| —highest | 1800 | 1200 | 2200 | 700 | | 1500 | 2000 | 1500 |
| **Blood Transfusion** ml | | | | | | | | |
| —median | 650 | 600 | 1000 | 600[b] | 750 | 1100 | 600 | 1000[b] |
| —lowest | 450 | 400 | 200 | 500 | | 900 | 450 | 375 |
| —highest | 2000 | 1100 | 1200 | 1200 | | 1250 | 1000 | 1500 |

[a]The blood loss in the 2 hr experiment is lower compared to the longer preservation groups (p = 0.03).
[b]Significantly different from OLT.
[c]Only one animal represented.

**Table 4** Causes of death after liver transplantation

| | *2 hr* | | *24 hr* | | *48 hr* | | *72 hr* | | *Total* |
|---|---|---|---|---|---|---|---|---|---|
| | *OLT* | *HLT* | *OLT* | *HLT* | *OLT* | *HLT* | *OLT* | *HLT* | |
| *Total* | *16* | *15* | *8* | *8* | *2* | *5* | *6* | *9* | *69* |
| Hemorrhage | 3 | 1 | 4 | 2 | 2 | 5 | 3 | 6 | 26 |
| Portal vein block[a] | | | | | | 3 | 1 | | 4 |
| Heart failure | | | 1 | 1 | | | | 1 | 3 |
| Cholangitis | 1 | | 1 | | | | | | 2 |
| Volvulus | 3 | 2 | | 1 | | | | | 6 |
| Rejection[b] | 2 | | | | | | | | 2 |
| Technical | | 4 | | | | | | 1 | 5 |
| Sacrificed | 3 | 5 | | | | | | | 8 |
| Others[c] | 2 | 2 | | 1 | | | | | 5 |
| Unknown | 2 | 1 | 3 | 2 | | | | | 8 |

[a]Total intrahepatic thrombosis of the portal venous system.
[b]Only if rejection was thought to be the direct cause of death.
[c]Pulmonary sepsis (n = 3), pneumothorax (n = 1), peritonitis (n = 1).

### DISCUSSION

The results from this study clearly show that the host liver in HLT also protects the recipient from the deleterious effects of long-term preserved grafts on hemostasis. We found a dramatic and sustained increase in t-PA activity after reperfusion in OLT, while the increase in t-PA in HLT was limited and transient. In OLT, the deterioration of t-PA activity was directly related to the duration of graft storage. In HLT this effect was much less evident, which demonstrated that the protective effect of the host liver on the development of hyperfibrinolysis also holds true with longer graft storage times. There are several explanations for the increase of t-PA after reperfusion. First, endothelial cells, an important source of t-PA, are more susceptible to cold hypoxia and reperfusion than parenchymal cells[21,22]. Free oxygen radicals have been implicated in this mechanism of graft injury[23,24]. The damaged endothelium may release t-PA directly into the circulation. Another theory is that the tissue damage leads to the attraction of inflammatory cells, including macrophages[25,26]. When activated, these cells are stimulated to produce cytokines (interleukin-1, tumor necrosis factor), which may actively interfere with coagulation and fibrinolysis[27]. Recently, it has been shown that pretreatment with cyclosporine, a powerful inhibitor of the function of macrophages, ameliorated the hemostatic changes after reperfusion in pigs, as measured by prothrombin times, platelet counts, and fibrinogen levels[28]. The initial increase in t-PA activity in HLT may be explained by a too abundant release of t-PA to be instantaneously cleared by the host liver. Comparison of t-PA activity levels in the portal vein inflow and the venous outflow revealed a gradient across the graft, which indicated that t-PA is also actively released by the graft. It was found that t-PA levels in the first hepatic outflow of the graft were higher in HLT than in OLT. A possible explanation is that the partial resection of the liver graft in HLT is responsible for this. This extra handling could make endothelial cells more liable to release t-PA[29]. It was notable that the high t-PA activity levels were only found in the first hepatic outflow from the 2 hr and 24 hr cold storage grafts and not in the 48 hr and 72 hr groups. Possibly, the endothelial cells in the latter group are damaged to such an extent that they no longer contain t-PA. Their contents, including t-PA, may have leaked into the preservation fluid, which is subsequently washed out during flushing before reperfusion. Despite this t-PA depletion, subsequent contact activation of the blood with the injured graft endothelium may evoke systemic t-PA release as suggested above. Probably, the same process occurred in HLT, but now the cytokines may be cleared by the host liver.

Fibrinogen levels, in turn, showed a serious decline in the *pre*reperfusion period in both OLT and HLT. There was no difference between OLT and HLT, which suggests that the changes are related to the surgical procedure in general and not to the anhepatic period. Previous investigators have suggested that fibrinogenolysis during the use of an external bypass might also contribute to the decrease in fibrinogen levels[15,30]. The latter is however likely to be of minor influence, as in HLT no signs of hyperfibrinolysis were present nor was a shunt used. Although part of the decline can be ascribed to hemodilution, there has to be another explanation as the hematocrit, a measure of hemodilution, did not equally decrease. Most likely a surgical induced activation of the coagulation system and extravasation of hemostasis proteins to the extravascular space are involved[29].

Similar to the fibrinolytic parameters, reperfusion induced a serious deterioration in coagulation parameters in OLT, whereas the changes in HLT remained limited. There was an apparent influence of the graft storage time. Presumably release of procoagulant factors from the preserved graft or local clotting activation on the damaged endothelium had initiated a consumption coagulopathy, as has been previously described. The present observations, however, show that HLT has also a protective effect on coagulation changes.

Remarkable effects of $PGE_1$ were found in preventing warm ischemic and reperfusion injury[31] in both experimental[32] and clinical[10] studies. We therefore implemented a trial of $PGE_1$ in our experiment with long-term preservation. As determined by fibrinolytic and coagulation tests, our study provides no evidence of a protection against hemostatic disturbances. As opposed to the multitude of positive studies on $PGE_1$, it is difficult to explain why we found no protective effect at all. Besides having included not enough animals to detect small differences, there is probably only a narrow margin of therapeutic efficacy, if it exists, between trivial and irreversible damage. Apparently, the upper limit is surpassed with 24 hr preservation of the porcine liver. In addition, prostaglandins can be assumed to counteract a physiological protective mechanism that secludes marginally functioning areas in the graft. This may well prove useful in retrieving reversibly injured parenchyma[33] but in contrast, it could promote the entrance into the bloodstream of deleterious substances originating from injured cells.

In conclusion, it was demonstrated that in pigs changes in hemostasis after long-term graft storage are less dramatic in HLT compared to OLT. This suggests that HLT also protects the recipient from the effects of long-term preserved grafts. Addition of $PGE_1$ to the preservation fluid had no effect on the hemostatic changes in both OLT and HLT. Although these experiments are performed in the presence of a healthy host liver (although deprived from portal blood after the transplantation), while in the clinical situation the host liver is diseased, some remaining liver function is always present. The results are indicative of the benificial role of the (remnant function of the) host liver on hemostasis.

*Acknowledgements*

The authors wish to thank the staff of the laboratory for surgical research for their endurance and proficient assistance in these experiments. Ms A.M. Bijma is gratefully acknowledged for the histocompatibility testing. We are indebted to Mr W.J. Kooreman, The Upjohn Company, The Netherlands, for his kind gift of prostaglandin $E_1$ used in these studies.

*References*

1. Kang, Y.G., Martin, D.J., Marquez, J., Lewis, J.H., Bontempo, F.A., Shaw, B.W. Jr., Starzl, T.E. and Winter, P.M. (1985) Intraoperative changes in blood coagulation and thrombelastographic monitoring in liver transplantation. *Anesth. Analg.*, **64**, 888–896
2. Von Kaulla, K.N., Kayne, H., Von Kaulla, E., Marchioro, T.L. and Starzl, T.E. (1966) Changes in blood coagulation before and after hepatectomy or transplantation in dogs and man. *Arch. Surg.*, **92**, 71–79
3. Blecher, T.E., Terblanche, J. and Peacock, J.H. (1968) Orthotopic liver homotransplantation. *Arch. Surg.*, **96**, 331–339
4. Böhmig, H.J. (1977) The coagulation disorder of orthotopic hepatic transplantation. *Semin. Thromb. Hemostas.*, **4**, 57–82
5. Porte, R.J., Bontempo, F.A., Knot, E.A.R., Lewis, H.J., Kang, Y.G. and Starzl, T.E. (1989) Systemic effects of tissue plasminogen activator-associated fibrinolysis and its relation to thrombin generation in orthotopic liver transplantation. *Transplantation*, **47**, 978–984
6. Dzik, W.H., Arkin, C.F., Jenkins, R.L. and Stump, D.C. (1988) Fibrinolysis during liver transplantation in humans: role of tissue-type plasminogen activator. *Blood*, **71**, 1090–1095
7. Homatas, J., Wasantapruek, S., Von Kaulla, K.N. and Eiseman, B. (1969) Clotting abnormalities following orthotopic and heterotopic transplantation of marginally preserved pig livers. *Acta Hepato-splenol.*, **2**, 14–27
8. Mieny, C.J., Homatas, J., Moore, A.R. and Eiseman, B. (1968) Limiting functions of a preserved liver homograft. *Gastroenterology*, **55**, 179–182
9. Porte, R.J., Blankensteijn, J.D., Knot, E.A.R., de Maat, M.P.M., Groenland, ThN. and Terpstra, O.T. (1991) A comparative study on changes in hemostasis in orthotopic and auxiliary liver transplantation in pigs. *Transplant Int.*, **4**, 12–17
10. Gaber, A.O., Thistlethwaite, J.R. Jr., Busse-Henry, S., Aboushloe, M., Emond, J.C., Rouch, D. and Broelsch, C.E. (1988) Improved results of preservation of hepatic grafts preflushed with albumin and prostaglandins. *Transplant Proc.*, **20**, 992–993
11. Todo, S., Nery, J.R., Yanaga, K., Podesta, L., Gordon, R.D. and Starzl, T.E. (1989) Extended preservation of human liver grafts with UW solution. *JAMA*, **261**, 711–714
12. Jamieson, N.V., Sundberg, R., Lindell, S., Claesson, K., Moen, J., Vreugdenhil, P.K. Wight, D.G., Southard, J.H. and Belzer, F.O. (1988) Preservation of the canine liver for 24–48 hours using simple cold storage with UW solution. *Transplantation*, **46**, 517–522
13. Bijnen, A.B., Dekkers-Bijma, A.M., Vriesendorp, H.M. and Westbroek, D.L. (1979) Value of the mixed lymphocyte reaction in dogs as a genetic assay. *Immunogenetics*, **8**, 287–297
14. Blankensteijn, J.D., Groenland, ThN., Baumgartner, D., Vos, L.P., Kerkhofs, L.G.M. and Terpstra, O.T. (1990) Intraoperative hemodynamics in liver transplantation comparing orthotopic with heterotopic transplantation in the pig. *Transplantation*, **49**, 665–668
15. Denmark, S.W., Shaw, B.W. Griffith, B.P. and Starzl, T.E. (1983) Venous-venous bypass without systemic anticoagulant in canine and human liver transplantation. *Surg. Forum*, **34**, 380–382
16. Tobimatsu, M., Konomi, K., Saito, S. and Tsumagari, T. (1985) Protective effect of prostaglandin $E_1$ on ischemia induced acute renal failure in dogs. *Surgery*, **98**, 45–52
17. Verheyen, J.H., Mullaart, E., Chang, G.T.G., Kluft, C. and Wijngaards, G. (1982) A simple, spectrophotometric assay for the extrinsic (tissue-type) plasminogen activator applicable to measurement in plasma. *Thromb. Haemostas.*, **48**, 266–269
18. Kluft, C., Brakman, P. and Veldhuijzen-Stolk, E.C. (1976) Screening of fibrinolytic activity in plasma euglobulin fractions on the fibrin plate. In: Davidson J.F., Samama, M.M., Desnoyers, P.C., eds. *Progress in chemical fibrinolysis and thrombolysis.* New York: Raven Press, pp. 57–65
19. Friberger, P., Knos, M., Gustavsson, S., Aurell, L. and Claeson, G. (1978) Methods for the determination of plasmin, antiplasmin and plasminogen by means of substrate S-2251. *Haemostasis*, **7**, 138–145

20. Clauss, A. (1957) Gerinnungsphysiologische Schnell Methode zur Bestimmung des Fibrinogens. *Acta Haematol.* (Basel), **17**, 237–246
21. Marzi, I., Zhong, Z., Lemasters, J.J. and Thurman, R.G. (1989) Evidence that graft survival is not related to parenchymal cell viability in rat liver transplantation. The importance of nonparenchymal cells. *Transplantation*, **48**, 463–468
22. Caldwell-Kenkel, J.C., Currin, R.T., Tanaka, Y. Thurman, R.G. and Lemasters, J.J. (1989) Reperfusion injury to endothelial cells following cold ischemic storage of rat livers. *Hepatology*, **10**, 292–299
23. De Groot, H. and Brecht, M. (1991) Reoxygenation injury in rat hepatocytes — mediation by $O_2$-/$H_2O_2$ liberated by sources other than xanthine oxidase. *Biol. Chem. Hoppeseyler*, **372**, 35–41
24. Southard, J.H., Marsh, D.C., McAnulty, J.F. and Belzer, F.O. (1987) The importance of $O_2$-derived free radical injury to organ preservation and transplantation. *Transplant Proc.*, **19**, 1380–1381
25. Takei, Y., Marzi, I., Kauffman, F.C., Currin, R.T., Lemasters, J.J. and Thurman, R.G. (1990) Increase in survival time of liver transplants by protease inhibitors and a calcium channel blocker, nisoldipine. *Transplantation*, **50**, 14–20
26. Nolan, J.P. (1981) Endotoxin, reticuloendothelial function and liver injury. *Hepatology*, **1**, 458–465
27. Nawroth, P.P., Handley, D. and Stern, D.M. (1986) The multiple levels of endothelial cell — coagulation factor interactions. In: Chesterman, C.N. (ed.) *Clinics in Haematology*, 293–321
28. Kim, Y.I., Kawano, K., Nakashima, K., Goto, S. and Kobayshi, M., (1991) Alleviation of 3.5 hour warm ischemic injury of the liver in pigs by cyclosporin pretherapy. *Transplantation*, **51**, 731–733
29. Hickman, R., Bracher, M., Pienaar, B.H. and Terblanche, J. (1991) Heparin as the cause of coagulopathy which may complicate grafting of the liver. *Surg. Gynecol. Obstet.*, **172**, 197–206
30. Rettke, S.R., Janossy, T.A., Chantigian, R.C., Burritt, M.F., Van Dyke, R.A., Harper, J.V., Ilstrup, D.M., Taswell, H.F., Wiesner, R.H. and Krom, R.A.F. (1989) Hemodynamic and metabolic changes in hepatic transplantation. *Mayo Clin. Proc.*, **64**, 232–240
31. Ueda, Y., Matsuo, K., Kamei, T., Kayashima, K. and Konomi, K. (1989) Protective effect of prostaglandin $E_1$ ($PGE_1$) on energy metabolism and reticuloendothelial function in the ischemically damaged canine liver. *Liver*, **9**, 6–13
32. Ueda, Y., Matsuo, K., Kamei, T., Ono, H., Kayashima, K., Tobimatsu, M. and Konomi, K. (1987) Prostaglandin $E_1$ but not $E_2$ is cytoprotective of energy metabolism and reticuloendothelial function in the ischemic canine liver. *Transplant Proc.*, **19**, 1329–1330
33. Greig, P.D., Woolf, G.M., Sinclair, S.B., Abecassis, M., Strasberg, S.M., Taylor, B.R., Blendis, L.M., Superina, R.A. Glynn, M.F.X., Langer, B. and Levy, G.A. (1989) Treatment of primary liver graft nonfunction with prostaglandin $E_1$. *Transplantation*, **48** 447–453

*(Accepted by S. Bengmark 23 April 1993)*

